# Trends in place of death in Peru, 2017–2024: a nationwide analysis

**DOI:** 10.1186/s13690-026-01872-9

**Published:** 2026-04-27

**Authors:** Brayan Miranda-Chavez, Karla Manrique-Hipólito, José Amado-TIneo, Rubí Paredes-Angeles, Alvaro Taype-Rondan

**Affiliations:** 1 Grupo de Estudios e Investigación en Educación Médica y Bioética de la FACSA, EDUCAB, Universidad Privada de Tacna, Tacna, Peru; 2 Servicio de Geriatría, Hospital Nacional Guillermo Almenara Irigoyen, EsSalud, Lima, Peru; 3 Unidad de Cuidados Paliativos, Subgerencia de Atención Domiciliaria PADOMI, EsSalud, Lima, Peru; 4 Facultad de Medicina, Universidad Nacional Mayor de San Marcos, Lima, Peru; 5 Departamento de Emergencias, Hospital Nacional Edgardo Rebagliati Martins, EsSalud, Lima, Peru; 6 Universidad Científica del Sur, Lima, Peru; 7 Unidad de Investigación para la Generación y Síntesis de Evidencias en Salud, Vicerrectorado de Investigación, Universidad San Ignacio de Loyola, Lima, Peru; 8 EviSalud - Evidencias en Salud, Lima, Peru

**Keywords:** Place of death, Palliative care, End-of-life care, Health inequalities, Peru, Population-based study

## Abstract

**Introduction:**

The place of death is recognized as a proxy for the quality and equity of end-of-life care. Understanding where people die provides insights into health system performance, social inequalities, and the availability of palliative services. In Peru, evidence on this topic remains scarce, and national patterns and determinants have not been previously characterized.

**Methods:**

We conducted a cross-sectional analytical study including all deaths registered in SINADEF from January 2017 to April 2024. Deaths were categorized as occurring at home, in a healthcare facility, or elsewhere. Sociodemographic, geographic, and contextual variables were analyzed, and the need for palliative care was estimated using the methodology of The Lancet Commission on Global Access to Palliative Care and Pain Relief. Associations were assessed using Poisson regression models with robust variance to estimate adjusted prevalence ratios (aPR) and 95% confidence intervals (CIs).

**Results:**

A total of 1,093,463 deaths were analyzed. Overall, 53.1% occurred in healthcare facilities, 41.3% at home, and 5.6% elsewhere. The proportion of home deaths increased over the study period (*p* < 0.001). Older age, lack of health insurance, lower educational level, lower socioeconomic status, Indigenous ethnicity, and residence outside Metropolitan Lima were associated with higher prevalence of home death. In contrast, individuals with estimated palliative care needs were less likely to die at home (aPR = 0.66; 95% CI: 0.65 to 0.66). Marked regional heterogeneity was observed, with home deaths ranging from 23.5% in Madre de Dios to 62.3% in Puno.

**Conclusions:**

More than four in ten deaths in Peru occur at home, with increasing trends over time and wide regional and social disparities. The findings suggest that dying at home often reflects structural inequities and limited access to institutional or palliative care rather than preference. Strengthening community- and home-based palliative services is crucial to ensure that end-of-life care aligns with patients’ needs and values.

**Supplementary Information:**

The online version contains supplementary material available at 10.1186/s13690-026-01872-9.

**Table:** 

Text box 1. Contributions to the literature
• This study provides the first nationwide analysis of place of death in Peru, using more than one million death certificates from 2017–2024.
• It reveals substantial regional and social inequalities, suggesting that dying at home may often reflect barriers to accessing institutional and palliative care rather than a genuine personal preference.
• By estimating the need for palliative care using the Lancet Commission method, it offers a novel framework to link end-of-life patterns with system capacity.
• The findings highlight the urgent need to strengthen community-based palliative care and reduce inequities in end-of-life services across low- and middle-income settings.

## Introduction

The place of death is widely regarded as an indirect indicator of the quality of end-of-life care [1]. Although previous studies suggest that most individuals prefer to die at home with adequate palliative support, this preference is often shaped or limited by clinical conditions, social circumstances, and structural constraints [2]. Consequently, the place of death represents a complex marker that reflects the interaction between personal needs and the health system’s capacity to meet them.

A systematic review including 27 studies and 14,920 participants found that 55% of patients with cancer preferred to die at home [3]. In accordance with this, a study in low- and middle-income countries reported wide variation in home deaths, with an overall prevalence of 62%, but ranging from 55% in Asia to 64% in South America and 72% in Africa [4]. In contrast, a multicountry population-based study in 12 Latin American nations showed that 31.3% of deaths occurred at home, whereas 57.6% took place in hospitals [5]. This heterogeneity in places of death illustrates differences in health system capacity, access to palliative care, socioeconomic conditions, and cultural preferences across regions.

Multiple determinants of place of death have been associated with a higher likelihood of dying at home include being married, access to multidisciplinary home-based palliative care, the presence of an informal caregiver, a diagnosis of cancer, and explicit patient or family preference for home death [6].

In Peru, scientific evidence on the place of death remains scarce. To date, only one published study conducted in Lima among patients with cancer receiving home-based palliative care reported that more than 80% of participants died at home [7]. This limited information contrasts with the growing global interest in the subject [8].

In this context, the present study aims to analyze national trends in place of death in Peru between 2017 and 2024, and to identify the key associated factors. By doing so, it seeks to provide evidence that can inform policies and strengthen health systems to deliver more appropriate, person-centered end-of-life care. However, place of death should be interpreted as an indirect indicator of access to and organization of end-of-life care, rather than a direct measure of quality. Therefore, the observed trends and associations may reflect territorial inequities and gaps in continuity of care.

## Methods

### Design and setting

We conducted a cross-sectional analytical study using data from the National Death Information System (SINADEF), covering all registered deaths from January 1, 2017, through April 30, 2024 [9].

For all deaths in Peru, death certificates are managed electronically through SINADEF, an online platform of the Ministry of Health (https://www.minsa.gob.pe/defunciones/). Causes of death are recorded using the International Classification of Diseases, 10th Revision (ICD-10), and the same ICD standard was used for the entire study period (2017–2024). Physicians access the system with a unique username and password to complete the certificate, which includes information about the deceased, the cause of death, and the certifying physician. Certificates must be completed immediately when death occurs in a healthcare facility, or within 24 h if the death occurs elsewhere. Prior to the implementation of SINADEF in 2017, death certificates were issued in physical format and subsequently digitized through local computer applications [10].

### Participants

We included all deaths registered in SINADEF between January 1, 2017, and April 30, 2024. Records lacking information on sex, age, or place of death were excluded.

### Data source and procedures

The database was retrieved from the National Open Data Platform of the Peruvian government [11], where it was freely available. This database was downloaded in CSV format and imported into STATA for statistical analysis.

### Variables

#### Place of death

Initially classified into six categories (workplace, home, healthcare facility, in transit, public road, and other). For analytical purposes, these were grouped into three categories: home, healthcare facility, and other (which included workplace, in transit, public road, and other).

It should be noted that the death certificate does not specify whether the “healthcare facility” corresponds to a public or private center, nor does it detail the specific type of facility (e.g., hospital, care home/long-term care facility, specialized palliative care unit). It only records the general category “healthcare facility.”

#### Sociodemographic and contextual variables

Sex (male, female), age (< 18 years, 18–59 years, 60–74 years, 75–89 years, ≥ 90 years), marital status (married/cohabiting or single/widowed/divorced), educational level (no education, initial [including incomplete primary], complete primary, complete secondary, complete higher, and not reported), ethnic group (Mestizo, Amazonian Indigenous, Andean Indigenous, Afro-descendant, Asian, unclassified), region (Lima, coast [excluding Lima city], highlands, jungle), year of death (pre-pandemic: 2017–2020; pandemic: 2021–2022; post-pandemic: 2023–2024), health insurance (Social secure [EsSalud], Armed Forces, Comprehensive Health Insurance [SIS], other, none), and monetary poverty level (five groups, with group 1 representing the highest incidence of poverty and group 5 the lowest), where each decedent was classified into one of the five departmental monetary poverty groups according to the department of residence recorded in SINADEF and INEI’s classification of departments with statistically similar levels of monetary poverty [12].

#### Palliative care need

Palliative care need was estimated using the methodology proposed by the Lancet Commission on Global Access to Palliative Care and Pain Relief, which operationalizes need in terms of serious health-related suffering (SHS) [13]. This approach identifies 20 specific clinical conditions that are considered to generate SHS and therefore require palliative care, and assigns to each condition a standardized palliative care need weight (w_i_), as described in the Commission’s report. This methodology is similar to that used by Leniz et al. [14].

In our study, we applied this framework to the multiple causes of death recorded in SINADEF. Each death certificate can include several ICD-10–coded causes of death (immediate, intermediate, and underlying). For analysis, these causes were organized as LCC_a to LCC_f. For every ICD-10 code in these fields, we assessed whether it corresponded to one of the 20 Lancet Commission conditions; if so, we assigned the corresponding need weight w_i_, and if not, the weight was set to zero. Because a single individual may present more than one qualifying condition, we used the maximum weight across all recorded causes (from LCC_a to LCC_f) as the best indicator of that person’s SHS burden.

At the individual level, a decedent was classified as “needing palliative care” if at least one of their causes of death had a weight greater than zero. Those whose causes of death all had a weight of zero were classified as not needing palliative care. At the population level, the overall proportion of deaths with palliative care need was calculated as the number of individuals classified as needing palliative care divided by the total number of deaths in the study period. The list of the 20 conditions, their corresponding ICD-10 codes, and the exact weights (w_i_) used in our calculations is provided in Supplementary Table S1.

### Data analysis

The descriptive analysis included absolute and relative frequencies for categorical variables, alongside means and standard deviations for continuous variables. Temporal trend analyses of the proportion of deaths was conducted using the Cochran-Armitage test.

Factors associated with the place of death (home vs. non-home; healthcare facility vs. non-healthcare facility) were assessed by estimating prevalence ratios (PRs) and 95% confidence intervals (CIs) from Poisson regression models with robust variance. Given the large sample size, even small differences could reach statistical significance without practical relevance [15]. To mitigate this, we predefined a 10% effect size threshold as meaningful. Accordingly, associations were considered relevant only when the PR was ≥ 1.10 or ≤ 0.90 in both crude and adjusted analyses.

### Ethics

Ethics approval and consent to participate: The study was approved by the Ethics Committee of the Faculty of Health Sciences at the Private University of Tacna (FACSA-CEI/133-08-2025) and was conducted in accordance with the ethical principles of the Declaration of Helsinki. 

## Results

### Descriptive Results

Between 2017 and 2024, the SINADEF database recorded 1,133,608 deaths. After excluding records lacking information on place of death (3,065), department (34,680), sex (108), country (2,105), or age (187), the final analytic sample comprised 1,093,463 deaths (96.5% of the initial records).

Among the study population, 53.1% died in health facilities, 41.3% at home, and 5.6% elsewhere. The median age at death was 72 (RIC 56 to 83) years, and 55.2% were male. Regarding educational level, 13.7% had no formal education and 26.4% had completed only primary education. In terms of health insurance coverage, 49.8% were affiliated with the Seguro Integral de Salud (SIS), whereas 14.3% lacked any form of insurance. Overall, 66.1% of the population was deemed to have required palliative care (Table 1).

**Table 1:** Characteristics of the study population and factors associated with place of death

	Total (column percentages sum to 100%)	Deaths at home (*n* = 353 326), *N* (%)	Deaths in health facilities (*n* = 580 529), *N* (%)	Deaths at home, aPR (95% CI)**	Deaths in health facilities RPa (95% CI)**
Age					
Children/adolescents (0–18 years)	50 412 (4.6)	12 046 (23.9)	32 731 (64.9)	Ref	Ref
Adults (18–59 years)	268 138 (24.5)	79 100 (29.5)	155 841 (58.1)	1.39 (1.36 to 1.41)	0.74 (0.74 to 0.75)
Older adults (60–74 years)	279 919 (25.6)	100 153 (35.8)	169 283 (60.5)	1.76 (1.73 to 1.79)	0.71 (0.70 to 0.71)
Elderly (75–89 years)	371 403 (34.0)	182 951 (49.3)	179 280 (48.3)	2.29 (2.25 to 2.33)	0.59 (0.59 to 0.60)
Oldest-old (≥ 90 years)	123 591 (11.3)	77 368 (62.6)	43 394 (35.1)	2.83 (2.78 to 2.88)	0.45 (0.45 to 0.45)
Sex					
Female	489 528 (44.8)	220 773 (45.1)	251 237 (51.3)	Ref	Ref
Male	603 935 (55.2)	230 845 (38.2)	329 292 (54.5)	0.95 (0.94 to 0.95)	1.00 (0.99 to 1.00)
Type of insurance					
Uninsured	156 715 (14.3)	82 737 (52.8)	52 736 (33.7)	Ref	Ref
EsSalud	334 400 (30.6)	103 067 (30.8)	221 009 (66.1)	0.52 (0.52 to 0.53)	1.90 (1.88 to 1.91)
Armed Forces	24 674 (2.3)	5 735 (23.2)	17 906 (72.6)	0.40 (0.39 to 0.41)	2.04 (2.02 to 2.06)
SIS	544 202 (49.8)	248 671 (45.7)	269 185 (49.5)	0.70 (0.70 to 0.71)	1.60 (1.59 to 1.61)
Others	33 472 (3.1)	11 408 (34.1)	19 693 (58.8)	0.57 (0.56 to 0.58)	1.78 (1.76 to 1.80)
Marital status					
Married/cohabiting/common-law partner	475 955 (43.7)	202 974 (42.7)	255 874 (53.8)	Ref	Ref
Single/divorced/widowed	613 429 (56.3)	246 671 (40.2)	323 066 (52.7)	1.00 (0.99 to 1.00)	0.99 (0.99 to 0.99)
Educational level					
Not reported	297 402 (27.2)	41 996 (14.1)	246 968 (83.0)	0.28 (0.27 to 0.28)	2.16 (2.14 to 2.18)
No education	149 914 (13.7)	93 088 (62.1)	50 887 (33.9)	Ref	Ref
Initial education	5 029 (0.5)	2 639 (52.5)	1 903 (37.8)	0.98 (0.96 to 1.01)	1.05 (1.01 to 1.08)
Primary education	288 115 (26.4)	166 850 (57.9)	106 464 (37.0)	0.97 (0.97 to 0.98)	1.13 (1.12 to 1.14)
Secondary education	236 979 (21.7)	99 605 (42.0)	114 986 (48.5)	0.85 (0.85 to 0.86)	1.35 (1.34 to 1.36)
Higher education	116 024 (10.6)	47 440 (40.9)	59 321 (51.1)	0.90 (0.89 to 0.90)	1.33 (1.32 to 1.34)
Ethnic group					
Afro-descendant	1 502 (0.1)	574 (38.2)	859 (57.2)	1.08 (1.02 to 1.14)	0.95 (0.91 to 0.99)
Asian descendant	1 721 (0.2)	670 (38.9)	988 (57.4)	0.92 (0.87 to 0.97)	1.05 (1.01 to 1.09)
Amazonian Indigenous	2 698 (0.3)	1 355 (50.2)	1 088 (40.3)	1.37 (1.31 to 1.42)	0.78 (0.74 to 0.81)
Andean Indigenous	53 843 (4.9)	34 945 (64.9)	14 914 (27.7)	1.18 (1.17 to 1.19)	0.72 (0.71 to 0.73)
Mestizo	967 609 (88.8)	385 406 (39.8)	530 814 (54.9)	Ref	Ref
Unclassified	62 910 (5.8)	27 140 (43.1)	30 466 (48.4)	1.04 (1.03 to 1.05)	0.95 (0.94 to 0.96)
Region					
Lima City (includes Callao)	371 583 (34.0)	138 205 (35.4)	236 594 (60.6)	Ref	Ref
Coast (excluding Lima/Callao)	390 231 (35.7)	152 885 (41.1)	198 003 (53.3)	1.08 (1.07 to 1.09)	0.91 (0.91 to 0.92)
Jungle	74 860 (6.9)	29 657 (39.6)	40 614 (54.3)	1.07 (1.06 to 1.08)	0.94 (0.94 to 0.95)
Highlands	256 789 (23.5)	130 871 (51.0)	105 318 (41.0)	1.11 (1.10 to 1.12)	0.86 (0.86 to 0.87)
Period					
Pre-pandemic (2017 to 2020)	323 393 (29.6)	123 829 (38.3)	176 292 (54.5)	Ref	Ref
During pandemic (2021 to 2022)	462 598 (42.3)	187 171 (40.5)	255 462 (55.2)	1.29 (1.28 to 1.30)	0.81 (0.80 to 0.81)
Post-pandemic (2023 to 2024)	307 472 (28.1)	140 618 (45.7)	148 775 (48.4)	1.37 (1.36 to 1.37)	0.76 (0.76 to 0.76)
Monetary poverty level					
Group 1 (lower)	169 241 (15.5)	86 191 (50.9)	71 177 (42.1)	Ref	Ref
Group 2	172 304 (15.8)	76 401 (44.3)	88 116 (51.1)	1.01 (1.00 to 1.02)	1.01 (0.99 to 1.02)
Group 3	608 274 (55.6)	238 556 (39.2)	338 288 (55.6)	0.98 (0.97 to 0.98)	1.01 (1.00 to 1.02)
Group 4	103 178 (9.4)	37 511 (36.4)	58 195 (56.4)	0.78 (0.77 to 0.79)	1.16 (1.15 to 1.17)
Group 5 (Ica)	40 466 (3.7)	12 959 (32.0)	24 753 (61.2)	0.76 (0.75 to 0.77)	1.17 (1.15 to 1.18)
Cause of death					
Neoplasms	74 345 (7.1)	48 882 (65.8)	24 429 (32.9)	1.70 (1.69 to 1.71)	0.57 (0.56 to 0.58)
Chronic diseases (excluding neoplasms)	705 833 (67.4)	264 810 (26.3)	422 571 (59.9)	Ref	Ref
Acute diseases	26 703 (2.5)	12 705 (47.6)	13 456 (50.4)	0.78 (0.77 to 0.79)	1.61 (1.59 to 1.63)
External causes of death	26 651 (2.5)	7002 (26.3)	7907 (29.7)	0.76 (0.74 to 0.77)	0.54 (0.53 to 0.55)
Ill-defined causes**	40 227 (3.8)	19 927 (49.5)	18 535 (46.01)	0.81 (0.80 to 0.82)	1.49 (1.47 to 1.51)
Others	172 975 (16.5)	73 073 (42.2)	76 322 (44.1)	0.77 (0.77 to 0.78)	1.35 (1.34 to 1.37)
Need for palliative care					
Yes	722 810 (66.1)	263 931 (36.5)	430 862 (59.6)	0.66 (0.65 to 0.66)	1.81 (1.79 to 1.83)
No	370 653 (33.9)	187 687 (50.6)	149 667 (40.4)	Ref	Ref

*Ref *Reference value

‘’ Ill-defined data: Specific symptoms or ICD-10 coding that begins with the letter “Z”

**Adjusted Poisson regression with robust variance; a model was generated with the variables: age group, sex, type of insurance, educational level, ethnic group, region, period, need for palliative care , and poverty group

### Trends in place of death

**Fig. 1:**
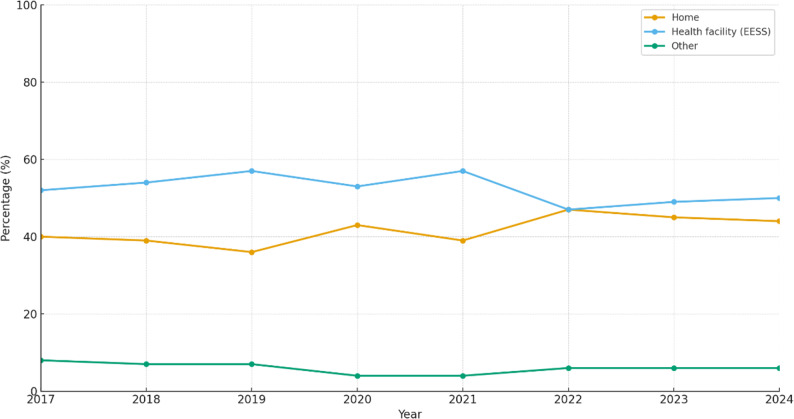
Trends in place of death by setting, Peru, 2017–2024

**Fig. 2:**
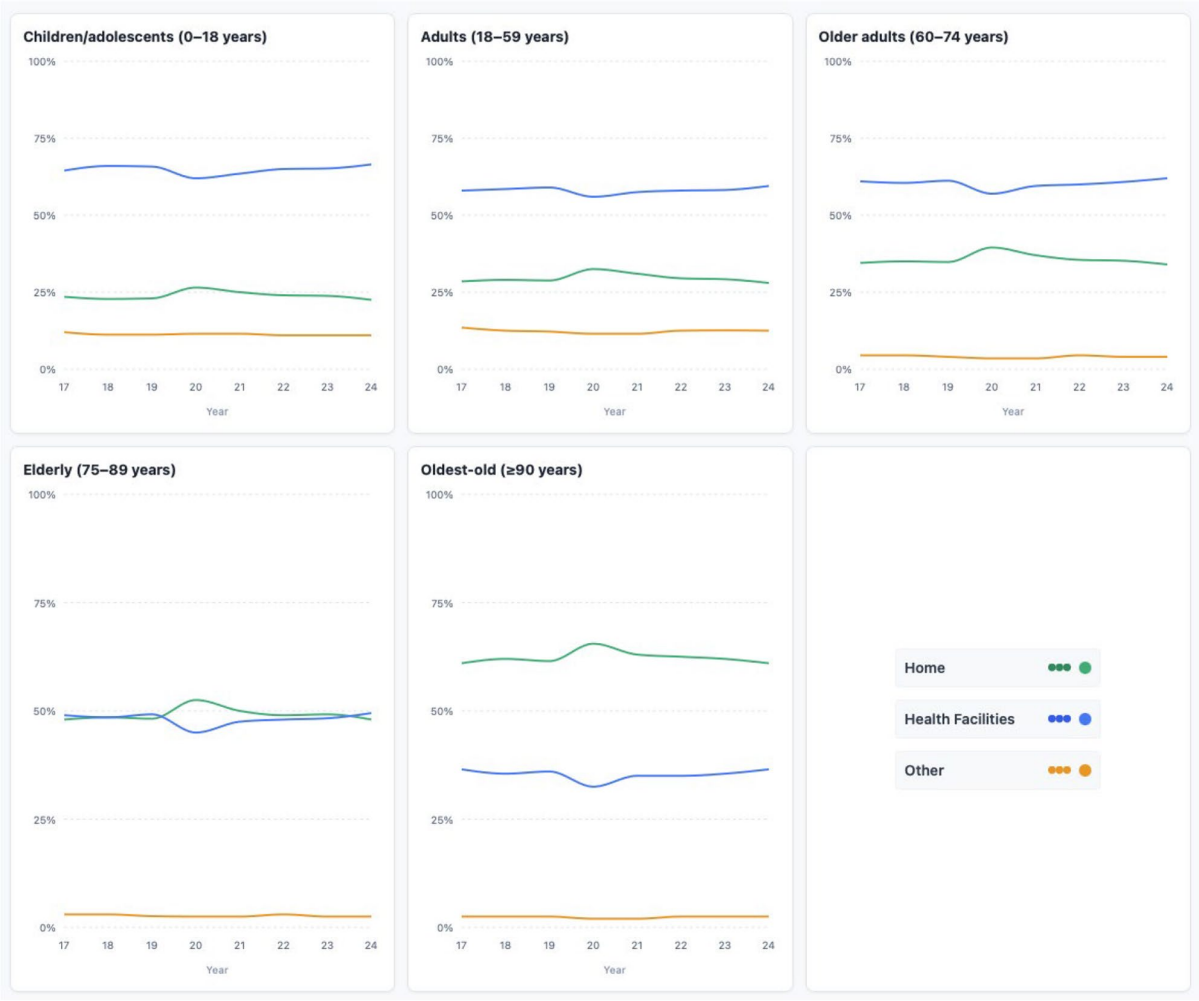
Trends in place of death by age group

Trends in place of death are shown in Fig. 1. There was a heterogeneous but overall increasing trend in deaths occurring at home (Cochran–Armitage test, *p* < 0.001), indicating that the proportion of home deaths rose over the study period. Conversely, a decreasing trend was observed for deaths in health facilities (*p* < 0.001). These patterns were consistent across all age groups (0–18, 19–59, and ≥ 60 years) (Fig. 2).

**Fig. 3:**
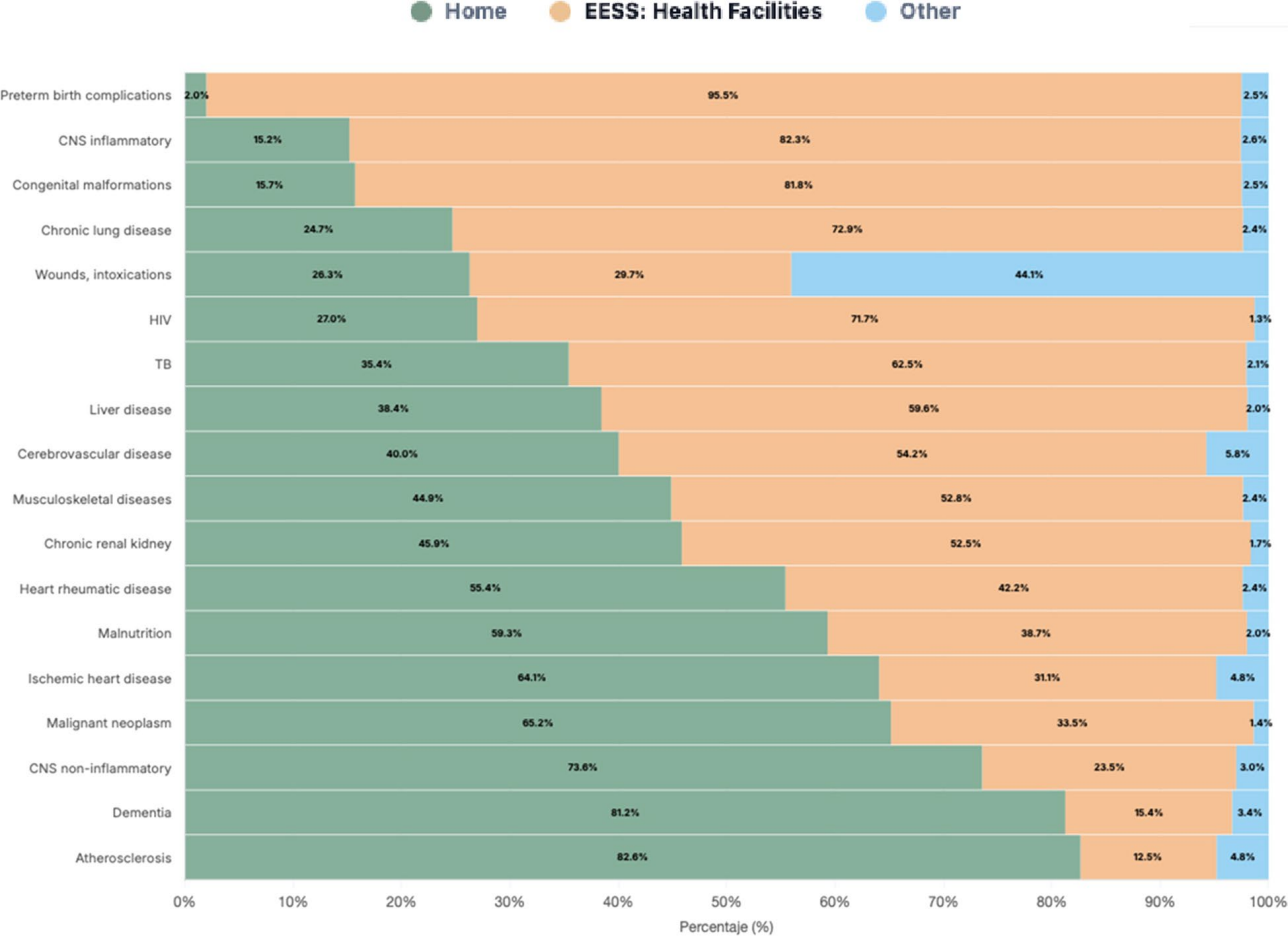
Place of death by cause of death among people with estimated palliative care needs, according to the Lancet method EESS: Health facilities

When examining deaths from conditions likely to require palliative care (per The Lancet Commission on Palliative Care and Pain Relief framework), the figure shows substantial heterogeneity in place of death. The share of home deaths range from 2.0% for preterm birth complications (with 95.5% occurring in EESS) to 82.6% for atherosclerosis, 81.2% for dementia, and 73.6% for non-inflammatory CNS disorders. At the lower end, home deaths were relatively uncommon for inflammatory CNS conditions (15.2%) and congenital malformations (15.7%), whereas several chronic conditions showed intermediate to high home-death proportions e.g., cerebrovascular disease (40.0%), chronic kidney disease (45.9%), ischemic heart disease (64.1%), and malignant neoplasms (65.2%). (Fig. 3).

**Fig. 4:**
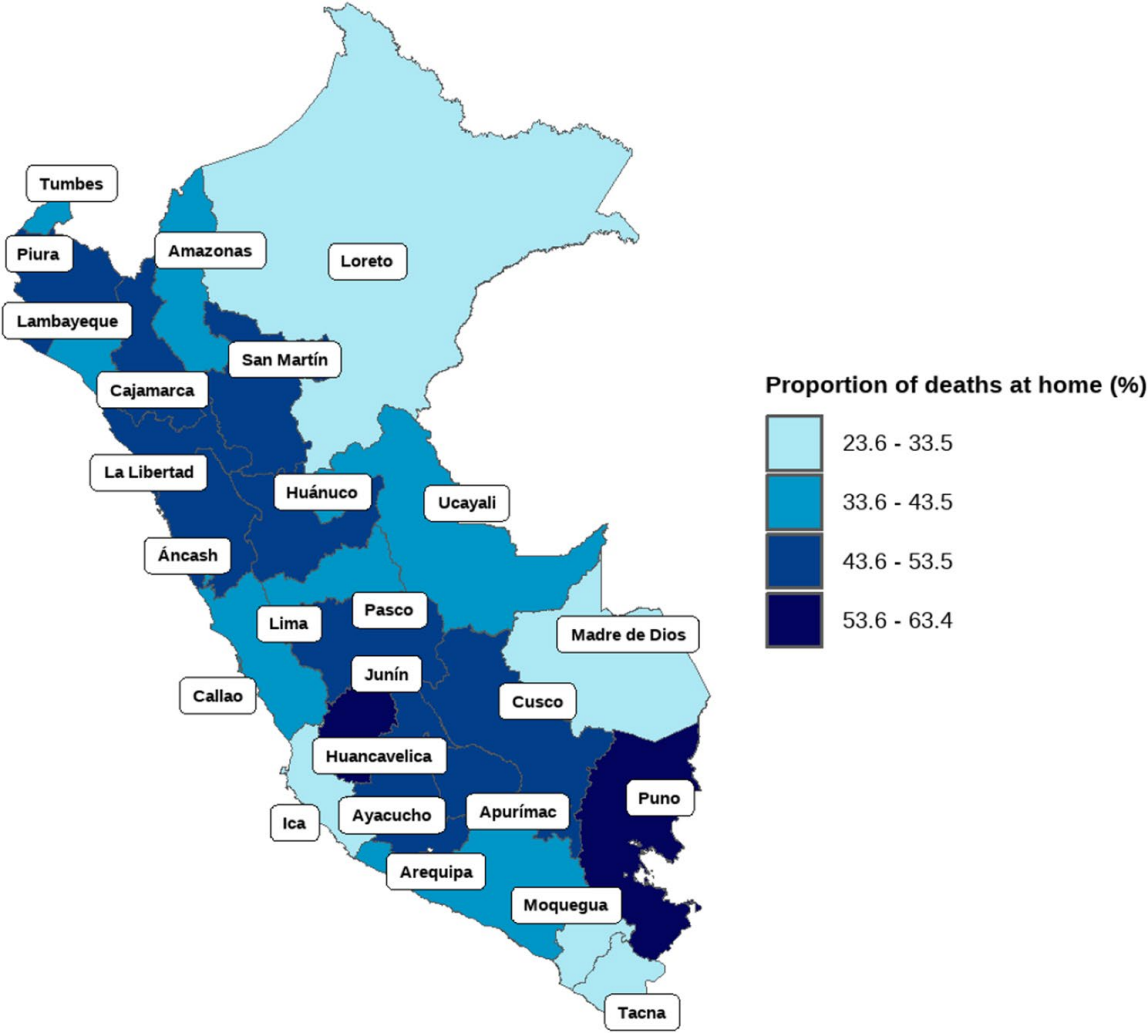
Proportion of deaths at home, by region

Regional differences were also evident. The proportion of home deaths was highest in Puno (62.3%), Huancavelica (57.3%), and Huanuco (51.0%), and lowest in Madre de Dios (23.5%) and Moquegua (27.5%) (Fig. 4).

### Factors associated with place of death

Older age was linked to a higher prevalence of dying at home, reaching its peak among the oldest adults (≥90 years) (aPR = 2.83; 95% CI: 2.78 to 2.88). 

Health insurance was inversely associated with dying at home: compared with the uninsured population, individuals covered by EsSalud showed the lowest prevalence of home death (aPR = 0.52; 95% CI: 0.52 to 0.53), followed by those affiliated with the Seguro Integral de Salud (SIS) (aPR = 0.70; 95% CI: 0.70 to 0.71). Notably, individuals covered by the Armed Forces health system had the highest proportion of deaths occurring in health facilities (72.6%) and more than twice the adjusted prevalence of health-facility death compared with the uninsured group (RPa = 2.04; 95% CI: 2.02 to 2.06) (Table 1).”

Regarding education, higher educational attainment was associated with a lower prevalence of dying at home. Completing secondary education (aPR = 0.85; 95% CI: 0.85 to 0.86) or having higher education (aPR = 0.90; 95% CI: 0.89 to 0.90) was linked to a reduced prevalence of home death. Similarly, belonging to Indigenous populations particularly Amazonian (aPR = 1.37; 95% CI: 1.31 to 1.42) and Andean groups (aPR = 1.18; 95% CI: 1.17 to 1.19) was associated with a higher prevalence of dying at home.

At the geographical level, deaths at home were more frequent outside Metropolitan Lima, with higher prevalence observed in the coastal regions (excluding Lima) (aPR = 1.08; 95% CI: 1.07 to 1.09), the jungle (aPR = 1.07; 95% CI: 1.06 to 1.08), and the highlands (aPR = 1.11; 95% CI: 1.10 to 1.12). Socioeconomic status also played an important role: individuals belonging to the wealthiest groups (Groups 4 and 5) were less likely to die at home than those in the poorest group (Group 1) (aPR = 0.78; 95% CI: 0.77 to 0.79 and aPR = 0.76; 95% CI: 0.75 to 0.77, respectively).va

Finally, cause of death and palliative care needs were significant factors. Deaths from neoplasms were more prevalence to occur at home compared with non-cancer chronic diseases (aPR = 1.70; 95% CI: 1.69 to 1.71). In contrast, individuals identified as requiring palliative care had a lower prevalence of home death (aPR = 0.66; 95% CI: 0.65 to 0.66) (Table 1).

## Discussion

### Summary of findings

We analyzed 1,093,463 deaths recorded in SINADEF between 2017 and 2024. Overall, 53.1% of deaths occurred in health facilities, 41.3% at home, and 5.6% elsewhere, with the proportion of home deaths showing an upward trend over time. The frequency of home deaths varied markedly across diagnostic categories and regions, reflecting both epidemiological and geographical heterogeneity.

A higher likelihood of dying at home was observed among individuals without health insurance, with lower educational attainment or lower socioeconomic status, and among residents outside Metropolitan Lima particularly in the coastal regions (excluding Lima), the highlands, and the jungle. Home deaths were also more common among Amazonian and Andean Indigenous populations compared with other ethnic groups, and the probability of dying at home increased sharply with age, peaking among those aged ≥ 90 years. An estimated 66.1% of the population required palliative care; among them, home deaths were less frequent (36.5% vs. 50.6%) and deaths in health facilities more frequent (59.6%).

### Trends in home deaths

In our national analysis, 41.3% of all deaths occurred at home, with a slight upward trend over the study period. Accordingly, a multicenter study conducted in 12 Latin American countries (2022) reported that 31.3% of deaths occurred at home and 57.6% in hospitals, with wide variation between countries (from 20% in Brazil to 67.9% in Guatemala). These differences were not explained by sociodemographic factors, suggesting that health policy and system organization play major roles [5].

In contrast, in Europe and North America, home deaths remain less common: a 2021 global analysis estimated them at 27.3% in high-income countries [16]. Nevertheless, there are signs of transition. in the United States and Europe. In the United States, the proportion of deaths occurring at home increased from 23.8% in 2003 to 30.7% in 2017 [17]. In England, one study documented an increase from 18.3% to 20.8% between 2004 and 2010 [18]. Moreover, an analysis for England and Wales (2018) projected that, if current trends persist, home and care-home deaths will rise substantially; without an expansion of community capacity, hospital deaths are likely to increase again [19].

Taken together, these findings suggest that the rise in home deaths is part of a wider global transition rather than a phenomenon restricted to high-income settings [20–23]. In this context, the proportion observed in our study (41.3%) is higher than the pooled Latin American estimate and the average reported for high-income countries, positioning Peru among the settings with a comparatively high frequency of home deaths. While this pattern may partly reflect strong cultural and familial preferences for dying at home, it may also signal structural barriers to accessing institutional and specialist palliative care, particularly in rural and socioeconomically disadvantaged areas [1, 22, 24, 25]. Interpreting these trends therefore requires careful consideration of whether home deaths reflect informed preference or constrained choice, and underscores the importance of monitoring place of death alongside indicators of quality of end-of-life care and coverage of community-based palliative services.

### Factors associated with dying at home

The prevalence of home deaths was higher among individuals without health insurance, those with lower educational attainment, lower socioeconomic status, and residents outside Metropolitan Lima. This pattern aligns with regional evidence. A 2022 multicountry study across 12 Latin American nations reported that individuals with less education and those living in rural areas were more likely to die at home a finding interpreted not as a fulfillment of end-of-life preferences, but rather as a consequence of limited access to institutional care and palliative services [5]. At the global level, a 2021 analysis found that home deaths were substantially more common in countries with lower health expenditure, limited health coverage, and greater rurality, supporting the hypothesis that the availability of hospital beds and formal care systems tends to shift deaths toward healthcare institutions [16].

In contrast, studies from high-income settings reveal a different dynamic. In England, a 2012 study reported an upward trend in home deaths but persistent inequities: individuals in more deprived areas were less likely to die at home, suggesting that when home-based and palliative services are available, those with higher social capital may be better able to achieve their “preferred place of death” [26]. Similarly, a 2011 Belgian study observed that deaths shifted primarily from hospitals to long-term care facilities rather than to private homes, underscoring the key role of the long-term care network in shaping place of death [22]. In Latin America, the 2018 Lancet Commission on Palliative Care and Pain Relief [13] highlighted a profound “access gap” in palliative services and opioid availability that disproportionately affects poor and rural populations, helping explain why uninsured and less-educated individuals, particularly outside capital cities such as Lima, are more likely to die at home out of necessity rather than choice.

A higher likelihood of dying at home was also observed among Amazonian and Andean Indigenous groups, consistent with international literature. In Australia, a 2007 study involving Indigenous patients, caregivers, and healthcare workers described a “clear articulation of Aboriginal peoples’ desire in rural and remote areas to die at home, connected to land and family” [27]. A 2017 Australian study similarly found that 46% of Indigenous people died at home [28], while research in New Zealand analyzing over 107,000 cancer-related deaths (2007–2018) showed that Māori patients were more likely to die at home, regardless of cancer type [29] .

This phenomenon may be explained by two interrelated factors: cultural worldview and healthcare accessibility. Culturally, Andean-Amazonian peoples often conceive death not as an end but as a spiritual transition, deeply intertwined with family and territory. Ethnographic work among Ashaninka communities of the Peruvian Amazon found that 83% of respondents had used traditional medicine in the past year, and 40% had never consulted a physician, suggesting a strong preference for home-based healing and traditional care practices [30]. In the same population, 57% reported being unable to see a doctor when needed—underscoring how access barriers may reinforce home deaths [31]. This stands in contrast to countries such as Canada, where a 2021 study reported that 56.1% of Indigenous deaths occurred in healthcare facilities, but 72.3% of this population had access to home care services [32], highlighting the role of service availability in shaping end-of-life trajectories.

The likelihood of dying at home also increased with age, a pattern consistent with Latin American data. The 2022 12-country study found that older adults were more likely to die at home, though hospitals remained the predominant setting [5]. In contrast, a 2020 study from Norway found that hospital deaths decreased with age, while deaths in nursing homes increased indicating that the expansion of long-term care facilities can shift deaths of very old adults away from the home. Similarly, in Japan, a 2020 study showed that the use of home care services was strongly associated with dying at home, suggesting that service availability modulates the relationship between age and place of death [33].

It is important to note that we observed a transitional pattern in the 75–89-year age group, which we interpret as a threshold at which multimorbidity and frailty intensify, increasing functional dependence and making transfers to hospital less feasible or less desirable. In this group, clinical and family decisions tend to shift toward comfort-oriented care after recurrent decompensations, and when available, home-based services (e.g., PADOMI within EsSalud) may facilitate dying at home by providing support outside the hospital [34]. In addition, health insurance coverage may operate through opposing pathways: greater coverage can facilitate access both to hospital care and to home-based programs, whereas limited coverage may increase deaths at home by creating barriers to timely access to institutional care. Finally, the rise in home deaths after the pandemic may be related to restrictions and avoidance of overwhelmed facilities, along with greater reliance on home caregiving effects that are likely concentrated among older adults.

Finally, in our study, only 36.5% of individuals with an estimated need for palliative care died at home. This contrasts with international evidence showing that receiving palliative care (not merely needing it) increases the likelihood of dying outside the hospital. For example, a Canadian study found that patients who received palliative care in the last six months of life were more likely to die at home than in care facilities (49.5% vs. 39.6%) [35], and a Brazilian study reported that participation in an outpatient palliative care program doubled the probability of dying at home or in hospice [36]. Conversely, population-based studies from Sweden and Germany illustrate persistent institutional dominance: in 2019, only about one-fifth of Swedes died at home [37], and in Germany in 2017, 21.3% of deaths occurred at home compared with 51.8% in hospitals [38].

These discrepancies shed light on our findings. First, the need for palliative care does not equate to receiving it where home-based teams are available, home deaths increase; where coverage is limited or hospital-centered, institutional deaths predominate [35, 39, 40]. Second, national policies and the local availability of palliative and long-term care services substantially explain the cross-country and regional variations observed in the place of death [31–35, 41, 42].

### Limitations and strengths

This study has some limitations. First, the information was obtained from a secondary administrative database (SINADEF), which entails the risk of underreporting and misclassification of key variables such as cause of death. In addition, it is important to consider that our study period includes the COVID-19 pandemic, which may have shifted a proportion of deaths toward the home setting in many countries.

Second, neither the underlying diagnosis nor the need for palliative care could be independently validated, as the analysis was based exclusively on the data recorded on death certificates. Likewise, it was not possible to specify the type of healthcare facility in which the patient died (primary, secondary, or tertiary level of care), nor to identify deaths occurring in nursing homes or long-term care institutions.

Third, the exclusion of 3.06% of deaths due to missing information on department represents an additional source of uncertainty. If these missing data were concentrated in specific geographic areas—particularly remote or rural regions with limited health infrastructure—our regional analyses might underestimate the true magnitude of geographic inequalities in place of death. For example, if deaths without department information occurred disproportionately in Amazonian or highland regions, where home deaths are more frequent, our estimates of regional disparities would be conservative. It should be noted that the early years of SINADEF implementation faced challenges in system reliability that may have differentially affected data completeness across regions [36].

This study also has important strengths. It used a nationwide database that includes all deaths recorded in the country since the implementation of SINADEF, ensuring high statistical power and representativeness. Furthermore, we applied an internationally standardized and validated methodology to estimate the need for palliative care, which facilitates comparisons with studies from other settings. Another strength is the inclusion of relevant sociodemographic and contextual variables, which enabled a comprehensive analysis of factors associated with the place of death within the Peruvian context.

## Conclusion

In Peru, nearly half of all deaths occur at home, with a modest but steady increase in recent years. However, this pattern reflects not only personal or cultural preferences but also structural inequities in access to healthcare and palliative services. Home deaths were more common among the uninsured, those with lower educational attainment, poorer and Indigenous populations, and residents outside Lima groups that often face barriers to institutional care. Strengthening community- and home-based palliative care, expanding health coverage, and reducing regional disparities are essential steps to ensure that the place of death reflects genuine choice and dignity rather than unmet healthcare needs. Finally, these findings support conducting a follow-up study that uses surveys to explore preferences for care and place of death.

## Supplementary Information

Supplementary Material 1.

## Data Availability

Information was obtained from SINADEF (Peru), and the final dataset is accessible for further studies.

## Abbreviations

SINADEF: National Death Information System (Peru)
EsSalud: Peruvian social security health insurance
SIS: Seguro Integral de Salud (Comprehensive Health Insurance, Peru)
SHS: Serious health-related suffering
ICD-10: International Classification of Diseases, 10th Revision
LMICs: Low- and middle-income countries
