# Supplementary material for: Trends in place of death in Peru, 2017–2024: a nationwide analysis
Source: Arch Public Health. 2026 Apr 27;84:93. doi: 10.1186/s13690-026-01872-9 (PMC13112657; doi:10.1186/s13690-026-01872-9)
Supplement: Supplementary file 1 — Supplementary Material 1. [file 13690_2026_1872_MOESM1_ESM.docx]

**Material Suplemmentary**

**Table S1: Lancet Commission on Global Access to Palliative Care and Pain Relief**

| **Condition Category** | **Conditions** | **ICD-10 Code** | **PC factor for Decedents** |
| --- | --- | --- | --- |
| Cancer | Malignant neoplasm, excluding leukemia | C00-C97 (except C91-C95) | 90% |
|  | Leukemia | C91-C95 | 90% |
| Cardiovascular Diseases | Cerebrovascular disease | I60-I69 | 65% |
|  | Rheumatic heart disease, cardiomyopathies, and heart failure | I05-I09, I10-I15, I42, I50 | Rheumatic fever (65%); Hypertensive heart disease (70%); Cardiomyopathy, myocarditis, endocarditis (40%); Chagas disease (30%) |
|  | Ischemic heart disease | I25 | 5% |
| Communicable Diseases | Hemorrhagic fevers | B33.4 | 100% |
|  | TB | A15-A19 | 100% |
|  | HIV | B20-B24 | 100% |
| Dementia | CNS inflammatory conditions | G00-G09 | Syphilis (G01 - 70%); Measles (G02 - 50%); Meningitis (G00, G03 - 30%); Encephalitis (G04, G05 - 30%); Trypanosomiasis (G028 - 100%) |
|  |  | F00-F04, G30-G32 | 80% |
| Other | CNS non-inflammatory conditions | G20-G26, G35-G37, G40-G41, G80-83 | Parkinson's disease (G20-G26 - 65%); Epilepsy (G40-G41 - 50%); Multiple sclerosis (G35-G37 - 100%); Other neurological conditions (G30-G32, G80-83 - 65%) |
|  | Chronic lung disease | J40-47, J60-70, J80-84, J95-99 | Chronic obstructive pulmonary disease (J40-J47 - 80%); Other (J60-J70, J80-J84, J95-J99 - 50%) |
|  | Liver disease | K70-K77 | 95% |
|  | Chronic kidney disease | N17-N19 | 45% |
|  | Preterm birth complications and birth trauma | P07, P10-P15 | Preterm birth complications (P07 - 75%); Birth asphyxia and birth trauma (P10-P15 - 40%) |
|  | Congenital malformations | Q00-Q99 | 60% |
|  | Wounds, intoxications, external causes | S00-S99, T00-T98, V01-Y98 | 30% |
|  | Atherosclerosis (not included in other categories) | I70 | 35% |
|  | Musculoskeletal diseases | M00-M97 | 70% |
|  | Malnutrition | E40-E46 | 100% |
